# Theoretical insights into a high-efficiency Sb_2_Se_3_-based dual-heterojunction solar cell

**DOI:** 10.1016/j.heliyon.2022.e09120

**Published:** 2022-03-16

**Authors:** Bipanko Kumar Mondal, Shaikh Khaled Mostaque, Jaker Hossain

**Affiliations:** Solar Energy Laboratory, Department of Electrical and Electronic Engineering, University of Rajshahi, Rajshahi, 6205, Bangladesh

**Keywords:** Sb_2_Se_3_, AgInTe_2_, Dual-heterojunction, High efficiency, TSA upconversion

## Abstract

Here, we manifest the design and simulation of an *n*-ZnSe/*p*-Sb_2_Se_3_/*p*^*+*^-AgInTe_2_ dual-heterojunction (DH) solar cell which exhibits a prominent efficiency. The performance of the solar cell has been assessed with reported experimental parameters using SCAPS-1D simulator by varying thickness, doping concentration and defect density in each layer. The proposed structure shows an efficiency of 38.6% with *V*_*OC*_ = 0.860 V, *J*_*SC*_ = 54.3 mA/cm^2^ and FF = 82.77%, respectively. Such a high efficiency close to Shockley-Queisser (SQ) limit of DH solar cell has been achieved as a result of the longer wavelength photon absorption in the *p*^*+*^-AgInTe_2_ back surface field (BSF) layer through a tail-states assisted (TSA) two-step photon upconversion phenomenon. These results indicate hopeful application of AgInTe_2_ as a bottom layer in Sb_2_Se_3_-based solar cell to enhance the cell performance in future.

## Highlights

• A novel *n*-ZnSe/*p*-Sb_2_Se_3_/*p*^*+*^-AgInTe_2_ DH solar cell has been simulated using SCAPS-1D.
• AgInTe_2_ BSF boosts PCE to 38.63% with *V*_*OC*_ = 0.86V and *J*_*SC*_ = 54.28 mA/cm^2^, respectively.
• AgInTe_2_ shows its potential as BSF in Sb_2_Se_3_-based DH solar cell in near future.

## Graphical abstract

**Figure:**
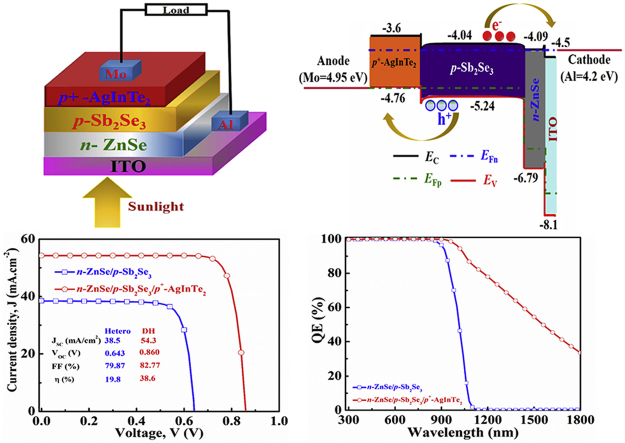

## 1 Introduction

In photovoltaic, a challenging part for researchers is obtaining a significant value of power conversion efficiency (PCE) along with selecting abundant low-cost and eco-friendly materials for large area production. The silicon solar cells are still dominating the present commercial markets although the highest PCE of the solar cells is just over 26% which has been reported in 2017 (Yoshikawa et al., 2017). Besides, Silicon's high melting point and minimal tolerance to defects in the manufacturing process necessitate a pricey processing environment (Steinmann et al., 2015). Additionally, higher production cost of wafer-based silicon solar cell and reduction of residential uses due to its fragility are the matter of concerns for traditional cells (Pathak et al., 2015; Bhopal et al., 2017). As a consequence, choice of alternative materials and simple fabrication process for evolution of cells have received enormous attention to the researchers (Tyagi et al., 2013).

Recently, different types of solar cells based on materials or fabrication process have received a great interest for capability of providing significant efficiency. For instance, Cadmium telluride (CdTe) and CIGS based solar cells have clinched around 22% of PCE (Polman et al., 2016; Green et al., 2018; Jackson et al., 2016). Researchers have showed that recombination losses in the solar cells are resulted from the inherited dangling bonds at grain boundaries of Si as well as CdTe, CdS and CIGS that degrade the cell performance (Zhou et al., 2015). However, toxicity of materials in these types of solar cells are notable concerns for fabrication in industry due to health hazard (Pathak et al., 2015). Besides, scarcity of such elements causes rise in overall production cost. Therefore, replacement of cadmium containing materials has become a key concern (Pathak et al., 2014). Another option can be the hybrid methylammonium lead halide perovskite (MAPbX_3_) based solar cell that has an efficiency of 20.1% but it exhibits insufficient tolerance to defects. More recently, the highest efficiency of 25.2% has been attained for perovskite solar cell by increasing charged carrier management (Yoo et al., 2021). Nevertheless, efficient perovskite solar cell uses harmful element lead (Pb) and exhibits lack of stability at low temperatures (Steinmann et al., 2015).

Antimony selenide, Sb_2_Se_3_ which has the simple single phase non-cubic orthorhombic chalcogenide structure can be a prominent absorber that can meet up all the constraints discussed above (Steinmann et al., 2015; Chen et al., 2017; Mavlonov et al., 2020; Guo et al., 2019; Shen et al., 2020). Such crystal structure tends to yield ribbon-like or layered morphologies that outcome with thoroughly anisotropic charge transport. However, Sb_2_Se_3_ is abundant on earth in the form of mineral subnite. Its thermal evaporation property at low temperature and vacuum (350 °C and ∼8 mTorr, respectively) further contributes in cost minimization. It has also been found to exhibit excellent photovoltaic absorbance due to band gap of 1.1–1.3 eV and high absorption coefficient (>10^5^ cm^−1^) at visible portion of solar spectrum. Besides, Sb_2_Se_3_ displays a good carrier mobility ($\approx 15\;cm^{2}V^{-1}s^{-1}$). Literature reports indicate that both acceptor and donor types of impurities have been used for doping into the Sb_2_Se_3_ thin films that can be used as absorber layers in thin film solar cells. Many investigations have been performed on p and n type dopants of Sb_2_Se_3_ thin films. Among them, Cu, Sn and Fe can be employed as p-type dopants whereas halogen (I, Br, Cl) group can be utilized as n-type dopants in Sb_2_Se_3_ thin films (Mavlonov et al., 2020; Stoliaroff et al., 2020).

In 2009, researchers came up with 0.66% photo conversion efficiency introducing Sb_2_Se_3_ based solar cell structure (Messina et al., 2009). An efficiency of 6–7% for Sb_2_Se_3_-based solar cells have been obtained in experimental works through tuning selenization parameters and grain boundary inversion, respectively (Chen et al., 2018; Tang et al., 2019). Furthermore, contemporary work came into light with growth of nanorod array with a photo conversion efficiency of 9.2% (Li et al., 2019). However, simulation works have also been performed to provide guidelines for further improvement of Sb_2_Se_3_-based solar cells. According to recent simulation studies, the efficiency of the Sb_2_Se_3_-based solar cell can be reached to 23–29% by employing CdS window layer and CuO and BaSi_2_ BSF layers (Li et al., 2019; Ahmed et al., 2021).

Zinc Selenide (ZnSe) can be an alternative solution to substitute toxic CdS in window layer for heterojunction solar cells. The efficiency of a photovoltaic cell relies on avoiding the recombination of photo generated carriers and such sidestepping is done by adding a thin window layer of a wide band gap material (Krause et al., 1994; Kale et al., 2006; Yater et al., 1996). ZnSe provides a wider band gap of 2.7eV than CdS of 2.4 eV (Ahmed et al., 2021). Another disadvantage is CdS window comes up with higher surface roughness and lower transmittance that limit the efficiency of the practical solar cells (Kim et al., 2010). To the contrary, polycrystalline nature and high refractive index of ZnSe provide higher transmittance in visible and ultraviolet region of solar spectrum (Jones and Woods, 1976; Green et al., 2010). Furthermore, low reaction to humidity of ZnSe is an additional advantage in industrial production (Yater et al., 1996).

Crossing the different cohort of solar modules, heterojunction solar cells has entered into fourth generation with a goal of overcoming the barriers of theoretical expectation (Sharma et al., 2015). A heterojunction is fabricated by attaching an n-type layer with a p-type layer of dissimilar materials. The absorption of photons in the absorber layer results photocurrent through the generation of electron-hole pairs which are separated by the space charges at the pn junction. However, the photo conversion efficiency of single heterojunction cells cannot go beyond the SQ limit due to (i) thermal relaxation loss by high energy photon absorbed by a low band gap substance and (ii) below-band gap absorption loss by excitation failure of low energy photon to a high band gap material (Malinkiewicz et al., 2014; Yamaguchi et al., 2018).

An improvement can be achieved with imparting comparatively higher band gap top layer and lower band gap back surface layer with absorbing material (Rong et al., 2018). Similar concepts have been found to overcome SQ limit for dual-heterojunction solar cells (Martí and Luque, 2015). AgInTe_2_ is a potential ternary chalcopyrite material exhibiting a band gap of 0.96–1.16 eV which is just below the band gap of Sb_2_Se_3_ (Benseddik et al., 2020; El-Korashy et al., 1999). Such a value along with high absorption coefficient (4×10^4^ cm^−1^ at an wavelength of 1095 nm) and also sub-band gap effects have brought it as a suitable candidate for back surface absorber layer (El-Korashy et al., 1999). Though several works have been found on AgInTe_2_ based solar cells, to the best of our knowledge, such a promising candidate has never been utilized as a BSF layer.

In this work, a novel Sb_2_Se_3_-based *n*-ZnSe/*p*-Sb_2_Se_3_/*p*^*+*^-AgInTe_2_ dual-heterojunction solar cell structure has been proposed and simulated with a view to provide longer wavelength light absorption and minimize photon loss in the back surface layer. The light trapping strategy and the reason of high efficiency have been illustrated with absorption of photon in Sb_2_Se_3_ absorber layer through tail-states assisted (TSA) two-step photon upconversion phenomenon in *p*^*+*^-AgInTe_2_ BSF as well as bottom absorber layer.

## 2 Design of proposed Sb_2_Se_3_-based dual-heterojunction and numerical simulation

### 2.1 Device structure

**Figure 1:**
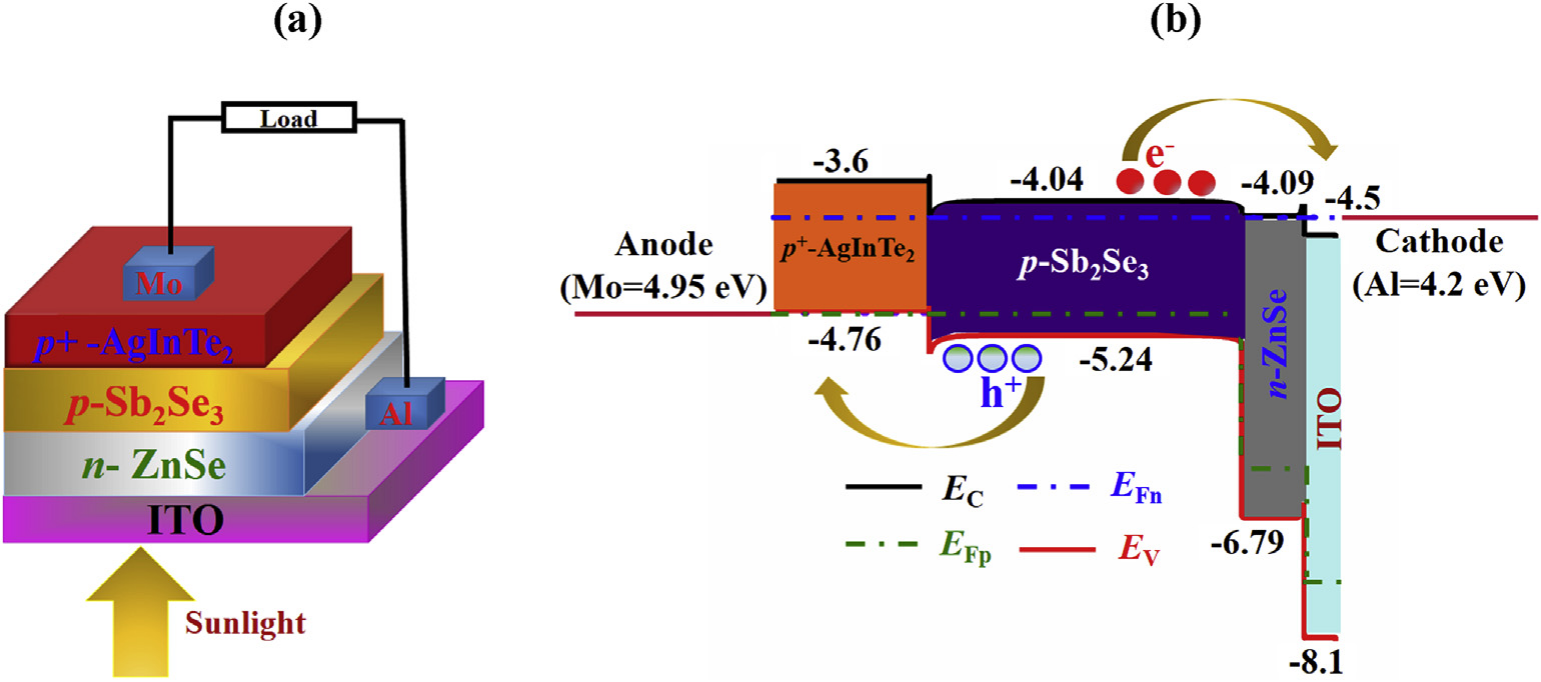
The (a) schematic block diagram and (b) energy band diagram with illumination of *n*-ZnSe/*p*-Sb_2_Se_3_/*p*^+^-AgInTe_2_ dual-heterojunction solar cell.

The schematic block diagram and illuminated energy band diagram of the proposed highly efficient Sb_2_Se_3_-based dual-heterojunction solar cell are visualized in Figure 1a and b, respectively. Herein, photons enter through the Indium tin oxide (ITO) coated glass substrate and n-type ZnSe window layer. The photons are then absorbed by p-type Sb_2_Se_3_ absorber layer. The E_C_ and E_V_ of ZnSe are 4.09 and 6.79 eV, respectively (Olopade et al., 2012; Samantilleke et al., 1998). On the other hand, the electron affinity and ionization potential of Sb_2_Se_3_ are 4.04 and 5.24 eV, respectively. Hence, it creates a possibility for n-type ZnSe and p-type Sb_2_Se_3_ to build a favorable pn junction. In addition, AgInTe_2_ is a semiconducting compound having a direct band gap of 0.96–1.16 eV which has already been reported as absorber layer with CdTe window combination (Benseddik et al., 2020). In this design, *p*^*+*^-type AgInTe_2_ has been employed to perform double role such as bottom absorber and back surface field (BSF) layer. Because of its high absorption coefficient (α) i.e sub-band gap absorption in the longer wavelength region, it is an efficient material as bottom absorber layer to absorb longer wavelength photons (El-Korashy et al., 1999; Vaidhyanathan et al., 1983). AgInTe_2_ has electron affinity and ionization potential of 3.6 eV and 4.76 eV, respectively making it a suitable candidate to form a pp^+^ junction with Sb_2_Se_3_. The quasi Fermi levels for electrons and holes are denoted as E_Fn_ and E_Fp_, respectively as depicted in Figure 1b. In AgInTe_2_, the E_Fn_ level lies above the VB edge whereas E_Fp_ level lies below the CB edge in ZnSe. Therefore, photo-generated electrons are moved towards the *n*-type window layer and blocked by BSF layer. Consequently, photo-generated holes are blocked by window layer and moved towards *p*^+^-type BSF layer. Therefore, anode and cathode can easily collect holes and electrons, respectively from absorber layer. In this design, earth abundant metals Al and Mo are used as cathode and anode, respectively.

### 2.2 Simulation model and physical parameters

The Sb_2_Se_3_-based *n*-ZnSe/*p*-Sb_2_Se_3_/*p*^+^-AgInTe_2_ dual-heterojunctionsolar cell was numerically simulated using a one-dimensional solar cell capacitance (SCAPS-1D) software developed by M. Burgelman et al. at the University of Gent, Belgium (Burgelman et al., 2004). This software analyzes solar cell structure by solving three basic equations of semiconductor i.e. Poisson's equation, the continuity equations for free holes and electrons and the drift-diffusion equation. The simulation was accomplished under the illumination of one sun with 100 mW/cm^2^ and the global air mass (AM) of 1.5G illumination spectrum at 300 K operating temperature. In this simulation, ideal values of series and shunt resistance were considered and radiative recombination coefficient was avoided. Gaussian energetic distribution was set for acceptor and donor defects for bulk layers and interface defects were also considered. Thermal velocity of 10^7^ cm/s was set for window, absorber and BSF layer and surface recombination velocity was taken 10^7^ cm/s for both metallic contacts. The absorption coefficient data for ZnSe, Sb_2_Se_3_ and AgInTe_2_ layer were collected from various literatures based on experimental works (El-Korashy et al., 1999; Adachi and Taguchi, 1991; El-Shair et al., 1991). It is noted that SCAPS software can automatically account the sub-band gap absorption effect on solar cell performance once the optical data are provided. The physical parameters for different layers used in this simulation are shown in Table 1, whereas interfaces parameters are shown in Table 2.

**Table 1:** Physical parameters of *n*-ZnSe/*p*-Sb_2_Se_3_/*p*^+^-AgInTe_2_ dual-heterojunction solar cell used in this simulation.

Parameters	ITO (Ahmmed et al., 2020)	*n*-ZnSe (Olopade et al., 2012; Samantilleke et al., 1998)	*p*-Sb_2_Se_3_ (Z. Q. Li et al., 2019)	*p*^+^-AgInTe_2_ (Benseddik et al., 2020; El-Korashy et al., 1999; Yang et al., 2017)
Layer type	Substrate	Window	Absorber	BSF
aThickness [μm]	0.05	0.1	1.0	0.5
Band gap, $E_{g}$ [eV]	3.6	2.7	1.2	1.16
Electron affinity, *χ* [eV]	4.5	4.09	4.04	3.6
Dielectric permittivity, *ε* [relative]	8.9	10	18	8.9
Effective CB density, *N*_*C*_ [cm^−3^]	2.2×10^18^	1.5×10^18^	2.2×10^18^	3.66×10^19^
Effective VB density, *N*_*V*_ [cm^−3^]	1.8×10^19^	1.8×10^19^	1.8×10^19^	1.35×10^19^
Electron mobility, $\;\mu_{n}$ [cm^2^ V^−1^ s^−1^]	50	50	15	1011
Hole mobility, $\mu_{p}$ [cm^2^ V^−1^ s^−1^]	10	20	5.1	887
aDonor concentration, *N*_*D*_ [cm^−3^]	1.0×10^21^	1.0×10^18^	0	0
aAcceptor concentration, *N*_*A*_ [cm^−3^]	1.0×10^7^	0	1.0×10^15^	3.5×10^19^
Defect type	Acceptor	Acceptor	Donor	Neutral/Donor
Energetic distribution	Gaussian	Gaussian	Gaussian	Gaussian
Reference for defect energy level, E_t_	Above the highest E_V_	Above the highest E_V_	Above the highest E_V_	Above the highest E_V_
Energy with respect to Reference [eV]	1.8	1.35	0.6	0.58
aPeak defect density, *N(t)* [eV^−1^ cm^−3^]	1.0×10^14^	1.0×10^13^	1.0×10^13^	1.0×10^13^
Characteristic energy [eV]	0.1	0.1	0.1	0.1
Electron capture cross section for defect [cm^2^]	10^−15^	10^−15^	10^−15^	10^−15^
Hole capture cross section for defect [cm^2^]	10^−15^	10^−15^	10^−17^	10^−15^

a) is a variable field.

**Table 2:** Interface parameters used in this simulation.

Parameters	ZnSe/Sb_2_Se_3_ interface	Sb_2_Se_3_/AgInTe_2_ interface
Defect type	Neutral	Neutral
Capture cross section for electrons [cm^2^]	10^−19^	10^−19^
Capture cross section for holes [cm^2^]	10^−19^	10^−19^
Energetic distribution	Single	Single
Reference for defect energy level, E_t_	Above the highest E_V_	Above the highest E_V_
Energy with respect to reference (eV)	0.6	0.6
Total defects (cm^−2^)	10^10^	10^10^

## 3 Results and discussion

### 3.1 Performance of Sb_2_Se_3_-based solar cells

#### 3.1.1 Role of Sb_2_Se_3_ absorber layer on PV parameters of n-ZnSe/p-Sb_2_Se_3_ solar cell

**Figure 2:**
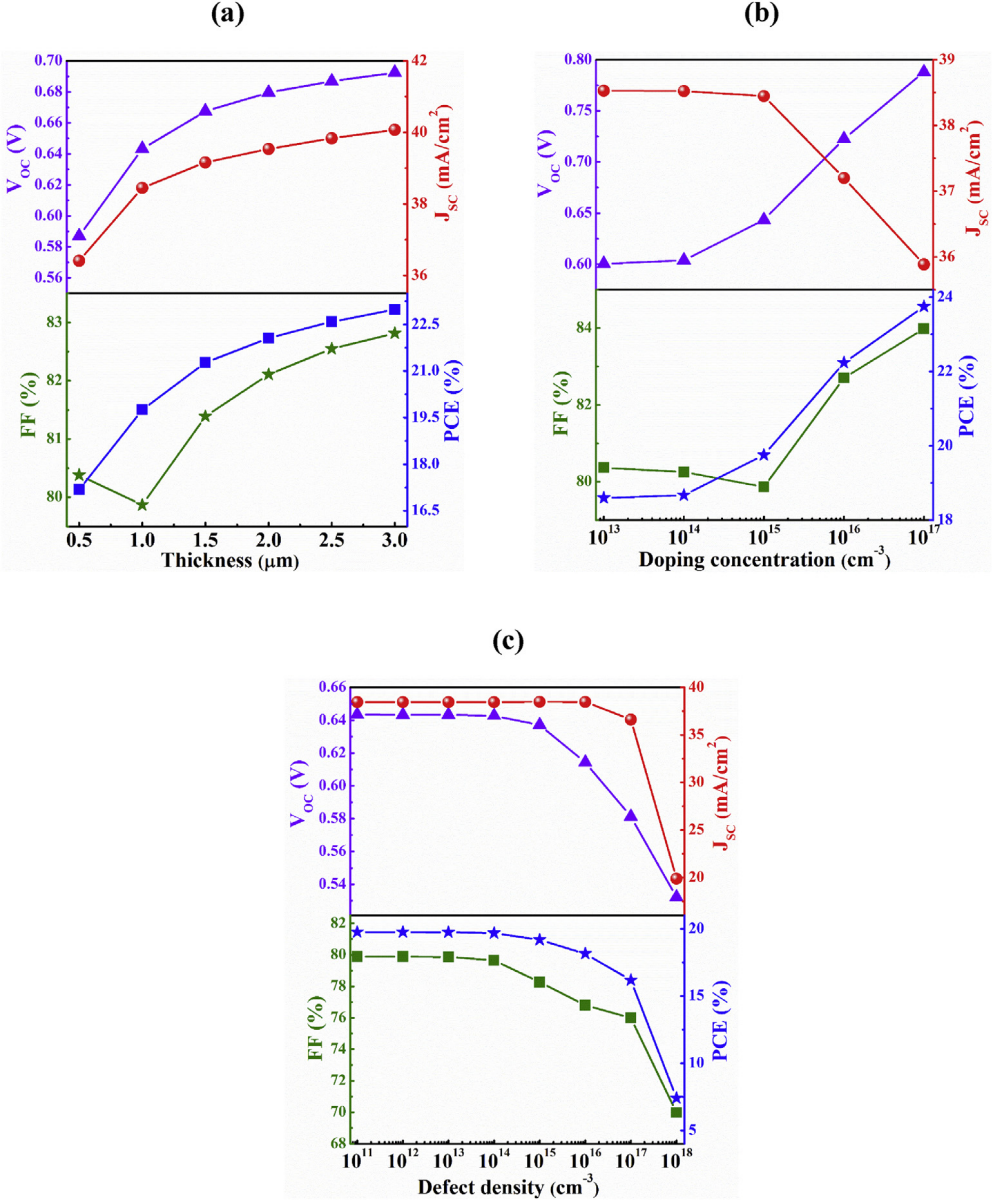
Performance dependency of n-ZnSe/p-Sb_2_Se_3_ solar cell on Sb_2_Se_3_ absorber layer parameters: (a) thickness, (b) doping concentration and (c) bulk defects.

In this section, the impacts of thickness, carrier concentration and bulk defects of absorber layer on the proposed Sb_2_Se_3_-based solar cell without BSF layer have been studied. Figure 2a depicts the dependency of solar cell performance on thickness of Sb_2_Se_3_ absorber layer considering 10^15^ and 10^13^ cm^−3^ as acceptor concentration and defect density, respectively. Here, we observe that as the thickness of Sb_2_Se_3_ absorber layer is increased, all photovoltaic (PV) parameters boost as well. The short circuit, *J*_*SC*_ has been risen up from 36.4 to 40.1 mA/cm^2^ with an increase in thickness from 0.5 to 3.0 μm. The increment of *J*_*SC*_ is reasonable because thicker absorber layer can create more electron-hole pairs (EHPs) through absorbing more photons incident on it (Hossain et al., 2020). At the same time, open circuit voltage, *V*_*OC*_ also increases with the thickness of the Sb_2_Se_3_ absorber layer. For instance, *V*_*OC*_ goes from 0.587 to 0.693 V at an enlargement of absorber thickness from 0.5 to 3.0 μm. The obtained *V*_*OC*_ of 0.680 V at 2.0 μm thickness of Sb_2_Se_3_ is consistent with the previous report (Kale et al., 2006). The fill factor (*FF*) and PCE have also ameliorated from 80.38 and 17.2 % to 82.82% and 22.9%, respectively at an increment of Sb_2_Se_3_ absorber from 0.5 to 3.0 μm. We consider 1.0 μm as the optimized thickness of the absorber Sb_2_Se_3_ layer to carry out further investigations.

The effect of acceptor concentration of Sb_2_Se_3_ absorber layer on PV parameters are shown in Figure 2b. The *V*_*OC*_ rises from 0.601 to 0.788 V when the carrier concentration is increased from 10^13^ to 10^17^ cm^−3^. However, *J*_*SC*_ is observed to downfall with the increase in carrier concentration. The recombination loss increases as a result of high doping concentration that affect the cell performance and a decrement of *J*_*SC*_ occurres from 38.5 to 35.9 mA/cm^2^ due to the enhancement of carrier concentration from 10^13^ to 10^17^ cm^−3^ (Hossain et al., 2020; Watahiki et al., 2016). However, as the series resistance gets lowered at higher carrier concentration, FF rises from 80.37 to 84%. Consequently, the PCE enhances from 18.60 to 23.75% depending on upgradation of *V*_*OC*_ and *FF*.

Defects play an important role in the performances of a solar cell. A number of investigations have been done on defects formation in Sb_2_Se_3_ for determining formation energy and transition levels that might be helpful for the improvement of the performance of Sb_2_Se_3_ based solar cells (Mavlonov et al., 2020; Savory and Scanlon, 2019; Stoliaroff et al., 2020). The variation of PV parameters with bulk defects of Sb_2_Se_3_ absorber layer is delineated in Figure 2c. To study the dependency of output parameters on defects of Sb_2_Se_3_ absorber layer, single donor type defects in the range from 10^11^ to 10^18^ cm^−3^ have been assumed. It is observed from the figure that the *J*_*SC*_ is about 38.45 mA/cm^2^ and is almost independent on bulk defects of absorber up to a defect of 10^16^ cm^−3^. However, if the bulk defect is further increased to 10^17^ cm^−3^, J_SC_ decreases to 36.6 mA/cm^2^ and the reduction of the photocurrent is about 1.8 mA/cm^2^. The photocurrent drastically plunges to 19.9 mA/cm^2^ at a defect of 10^18^ cm^−3^ owing to the increase in recombination current with defects (Kuddus et al., 2021b). The other parameters are also affected by the bulk defects. The Shockley-Read-Hall (SRH) recombination becomes the dominant recombination at high defects resulting inflation in the reverse saturation current and consequently *V*_*OC*_ of the device reduces (Moon et al., 2020). Here, The *V*_*OC*_ drops from 0.644 to 0.532 V when the defect density is raised from 10^11^ to 10^18^ cm^−3^. The *FF* also drops from 79.89 to 69.98% due to defects of absorber layer. As all the parameters gets reduced due to the increment of bulk defects of Sb_2_Se_3_ layer, the PCE of the solar cell also drastically turns down from 19.8% at defects of 10^11^ cm^−3^ to 7.4% at defects of 10^18^ cm^−3^. We have considered 10^13^ cm^−3^ as reasonable optimized defects for Sb_2_Se_3_ absorber layer.

The maximum efficiency of Sb_2_Se_3_-based solar cell without BSF layer is found to be about 23.8% with a high acceptor concentration of 10^17^ cm^−3^ and a thickness of 1.0 μm. Moreover, it has been observed that ZnSe window layer has negligible effects on the output parameters. Therefore, the optimized PCE of the Sb_2_Se_3_ solar cell without BSF layer is 19.8% with acceptor concentration and thickness of 10^15^ cm^−3^ and 1.0 μm, respectively.

#### 3.1.2 Role of Sb_2_Se_3_ absorber layer on quantum efficiency of n-ZnSe/p-Sb_2_Se_3_ solar cell

**Figure 3:**
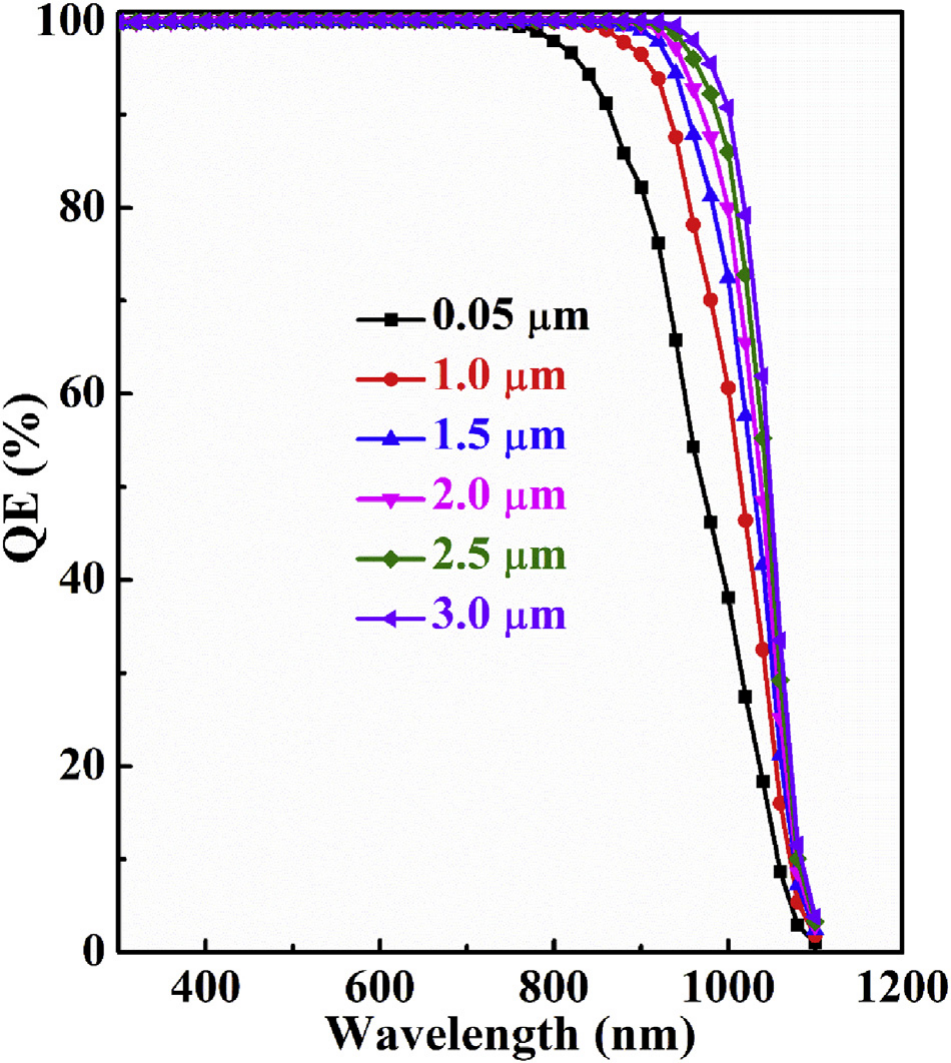
Simulated QE dependence on Sb_2_Se_3_ absorber layer thickness of n-ZnSe/p-Sb_2_Se_3_ single-heterojunction solar cell.

The quantum efficiency (QE) is a function of light wavelength (λ) which can be defined as the ratio of charge carriers produced by a solar cell to the number of incident photons on that cell (Hossain et al., 2021b; Moon et al., 2020). Figure 3 represents the simulated QE with respect to thickness of Sb_2_Se_3_ absorber layer of *n-*ZnSe/*p-*Sb_2_Se_3_ single heterojunction solar cell. It is noticed in the figure that QE improves gradually with the extension of Sb_2_Se_3_ absorber layer thickness as thicker absorber layer can capture more photons. Then all the curves associated different thickness downfall towards 0% at a particular higher wavelength when photon energy (hν) becomes lower than band gap (*E*_*g*_) energ*y* of Sb_2_Se_3_ absorber layer.

### 3.2 Sb_2_Se_3_-based solar cell with AgInTe_2_ BSF layer

#### 3.2.1 *Role of AgInTe*_*2*_*layer on PV parameters of n-ZnSe/p-Sb*_*2*_*Se*_*3*_/*p*^*+*^*-AgInTe*_*2*_*dual-heterojunction solar cell*

**Figure 4:**
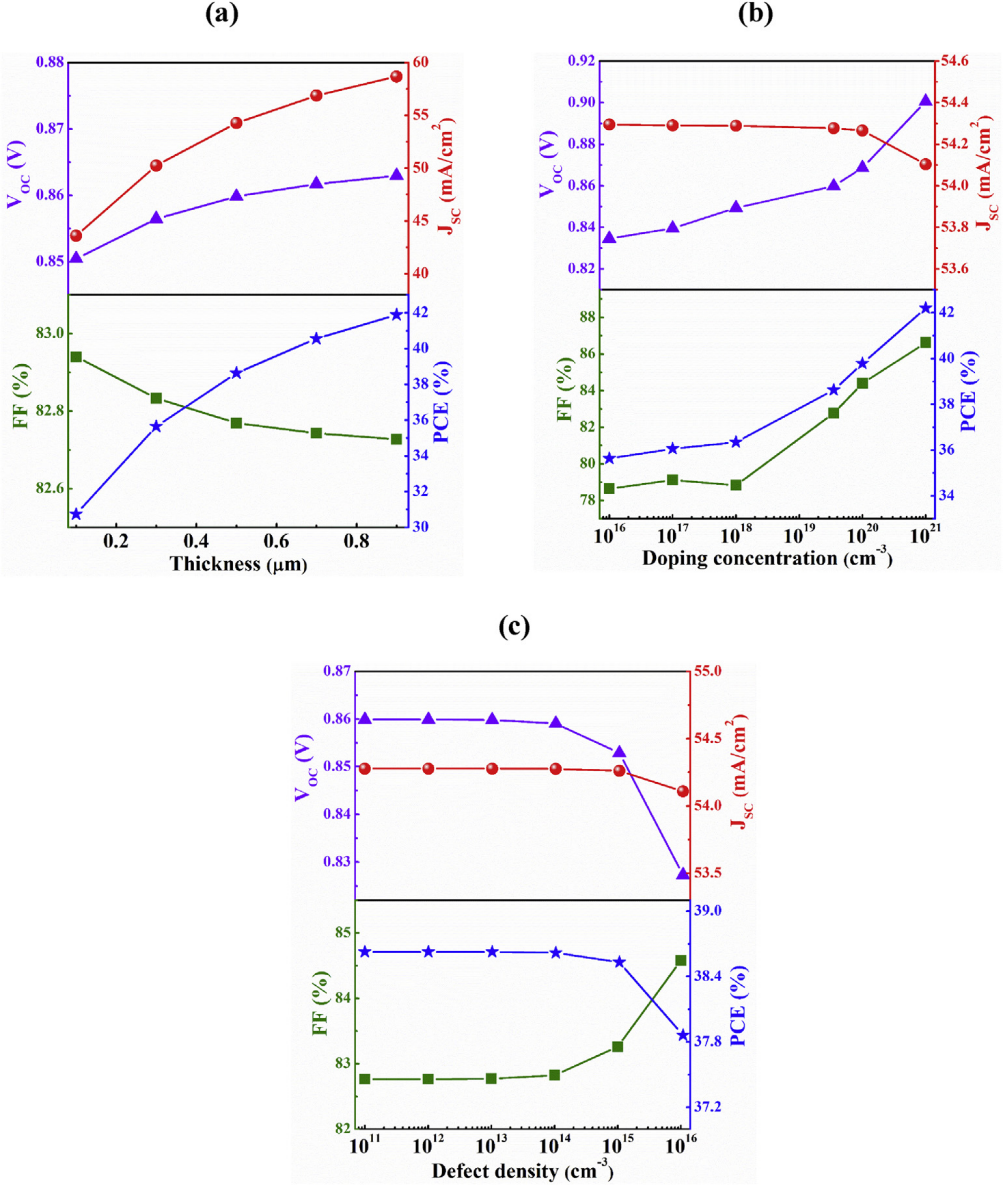
Dependency of the performance of *n-*ZnSe*/p-*Sb_2_Se_3_/*p*^+^-AgInTe_*2*_ dual-heterojunction solar cell on AgInTe_2_ BSF layer parameters: (a) thickness, (b) doping concentration and (c) bulk defects.

In this section, the influences of AgInTe_2_ layer on the designed *n-*ZnSe*/p-*Sb_2_Se_3_/*p*^+^-AgInTe_*2*_ dual-heterojunction structure have been explored. The thickness, doping concentration, and bulk defects of the AgInTe_2_ BSF layer have been varied in order to investigate the impact of this BSF layer as displayed in Figure 4.

Figure 4a exhibits the thickness dependent PV parameters of *n-*ZnSe*/p-*Sb_2_Se_3_/*p*^+^-AgInTe_2_ dual-heterojunction solar cell. As observed, all of the output parameters significantly improve on account of employing AgInTe_2_ layer that performs dual role as BSF and bottom absorber layer. Here, *J*_*SC*_ escalates from 38.5 to 54.3 mA/cm^2^ with an addition of 0.5 μm thick AgInTe_2_ layer. When the thickness of AgInTe_2_ is extended from 0.1 to 0.9 μm, *J*_*SC*_ enhances from 43.6 to 58.7 mA/cm^2^. The significant growth of *J*_*SC*_ relies on the absorption of longer wavelength photons by AgInTe_2_ layer through Tail-States-Assisted (TSA) two-steps photon upconversion process (Mostaque et al., 2022; Mondal et al., 2021; Kuddus et al., 2021a). In TSA upconversion process, two low-energy i.e. sub-band gap photons are absorbed in a sequence by Urbach tail-states of materials which provide extra EHPs. A material with preferable band gap, doping concentration and high absorption coefficient in longer wavelength region could result TSA upconversion process (Mostaque et al., 2022; Mondal et al., 2021; Hossain et al., 2021a, 2022). The detail qualitative discussion of the TSA upconversion process can be perceived in other work (Mostaque et al., 2022). Fortunately, AgInTe_2_ is a chalcopyrite material comprising a band gap of 1.16 eV (Malinkiewicz et al., 2014), high absorption coefficient of 10^3^ cm^−1^ in the wavelength of 1800 nm (obtained from SCAPS extrapolation utilizing absorption coefficient data from experimental work) (Benseddik et al., 2020; El-Korashy et al., 1999) and also possesses high carrier concentration of 3.66 × 10^19^ cm^3^ (Yang et al., 2017). All of these factors make AgInTe_2_ a suitable candidate to participate in a TSA upconversion process. The *V*_*OC*_ also improves from 0.640 to 0.840 V due to insertion of 0.5 μm AgInTe_2_ layer. The *V*_*OC*_ gradually enhances from 0.851 at 0.1 μm to 0.863 V at 0.9 μm thickness of AgInTe_2_. The insertion of AgInTe_2_ layer produces high built-in potential in the Sb_2_Se_3_/AgInTe_2_ interface which contribute to the enhancement of the V_OC_ (Hossain et al., 2021b; Mondal et al., 2021). The FF appears to be almost constant as a function of the thickness of AgInTe_2_. As *J*_*SC*_ and *V*_*OC*_ both rises with varying depth from 0.1 to 0.9 μm, a negligible change from 82.94 to 82.73% has been observed in FF. Eventually, the insertion of AgInTe_2_ BSF layer enriches the PCE from 30.8 to 41.9% as the width of BSF layer is expanded from 0.1 to 0.9 μm owing to the excessive gain in current and voltage. An enhancement of PCE from 19.8 to 38.6% has been observed at 0.5 μm thickness of AgInTe_2_ BSF layer.

Figure 4b delineates the performances of the proposed Sb_2_Se_3_-based DH solar cell structure with the variation of acceptor concentration (N_A_) of AgInTe_2_ layer from 10^16^ to 10^21^ cm^−3^ while maintaining the other parameters unchanged. It is observed from figure that the *J*_*SC*_ is almost constant up to the concentration of 10^20^ cm^−3^. It slightly falls from 54.3 to 54.1 mA/cm^2^ due to high carrier concentration which may happened due to the parasitic free carrier absorption or high doping may have negative effect in TSA upconversion process in the BSF layer (Kuddus et al., 2021a). The *V*_*OC*_ escalates from 0.835 to 0.901 V with increasing acceptor concentration from 10^16^ to 10^21^ cm^−3^of AgInTe_2_ layer. Since the built-in potential rises with additional N_A_, the enhancement of *V*_*OC*_ with doping concentration is reasonable since higher built-in potential will the reduce recombination current (Hossain et al., 2020; Mostaque et al., 2022). This trend is also evident in *FF* and PCE which elevate from 78.65 and 35.7% to 86.62 and 42.2%, respectively for the increase of carrier concentration from 10^16^ to 10^21^ cm^−3^.

To investigate the influences of defect density of AgInTe_2_ BSF layer, on Sb_2_Se_3_-based double-heterojunction solar cell, neutral/donor types of defects have been assumed and varied it from 10^11^ to 10^16^ cm^−3^ considering other parameters same as specified in Tables 1 and 2. The variation of photovoltaic parameters with bulk defects of AgInTe_2_ layer is depicted in Figure 4c. The *J*_*SC*_ shows almost constant behavior up to a defect level of 10^15^ cm^−3^ but then it has negligible decrement because of higher defects which obstruct to generate EHPs. The *V*_*OC*_ also drops from 0.860 to 0.827 V owing to the increase of defect level from 10^11^ to 10^16^ cm^−3^. Since, both *J*_*SC*_ and *V*_*OC*_ fall with increasing bulk defects of AgInTe_2_ layer, *FF* is found to improve from 82.76 to 84.58%. The PCE has also decreased from 38.6% at defect density of 10^11^ cm^−3^ to 37.9% at defect density of 10^16^ cm^−3^. Hence, utilizing AgInTe_2_ as BSF as well as bottom absorber layer in Sb_2_Se_3_ solar cell with reasonable defects, it is possible to attain the Shockley-Queisser (SQ) efficiency limit for a dual-heterojunction solar cell.

#### *3.2.2* *Role of AgInTe*_2_*layer on quantum efficiency of n-ZnSe/p-Sb*_*2*_*Se*_*3*_/*p*^+^-*AgInTe*_*2*_*dual-heterojunction solar cell*

**Figure 5:**
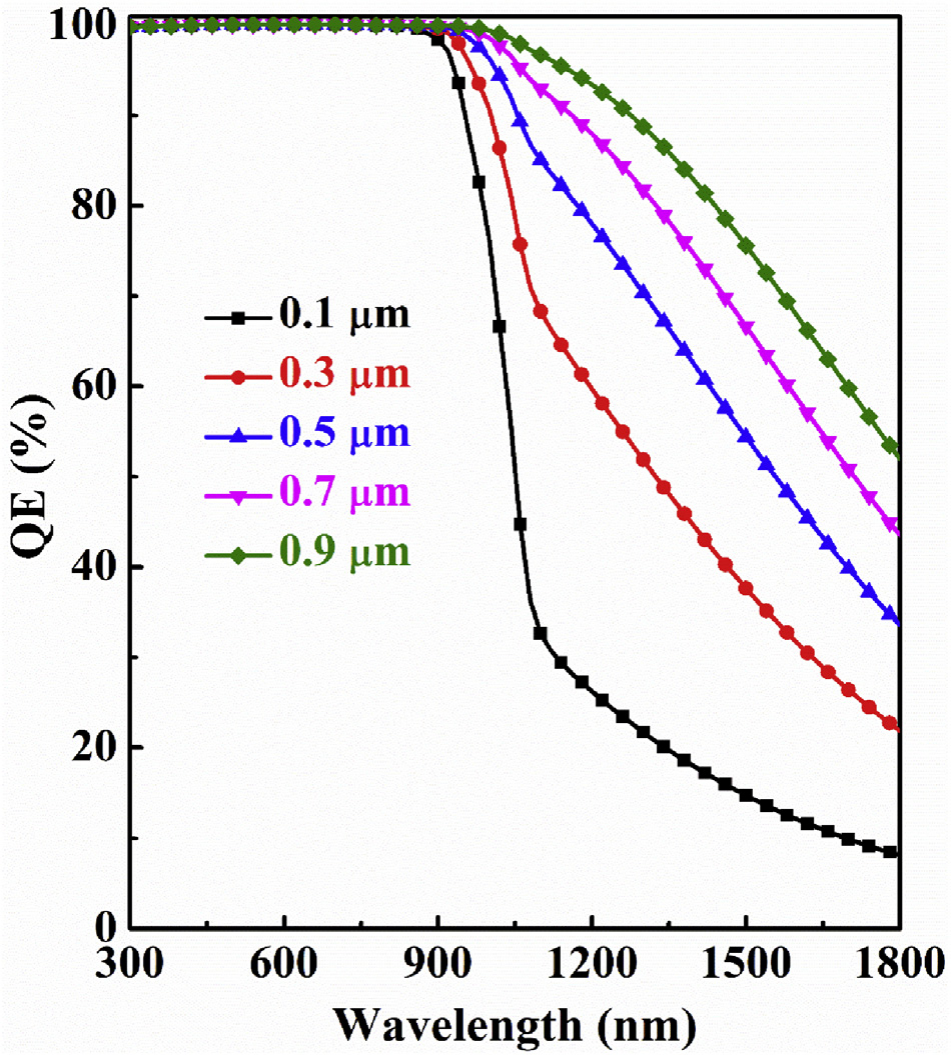
Simulated QE dependence of n-ZnSe/p-Sb_2_Se_3_/p^+^-AgInTe_2_ dual-heterojunction solar cell on the thickness of AgInTe_2_layer.

Figure 5 represents the simulated QE as a function of light wavelength for Sb_2_Se_3_-based dual-heterojunction solar cell with a varying thickness of AgInTe_2_ layer. It is noticed that, QE increases dramatically as the thickness of the AgInTe_2_ layer is gradually incremented from 0.1μm to 0.9 μm. In the longer wavelength range, for example, at 1800 nm, this device has a QE of over 30% at 0.5 m thickness, whereas 0% QE in absence of the AgInTe_2_ layer as observed in Figure 3. It clarifies the ability of AgInTe_2_ layer to capture longer wavelength photons as demonstrated by the TSA two-steps photon upconversion process in which the creation of additional EHPs plays a significant role behind this augmentation (Mostaque et al., 2022; Mondal et al., 2021; Kuddus et al., 2021a).

### 3.3 The optimized performance of single and dual-heterojunction cells

**Figure 6:**
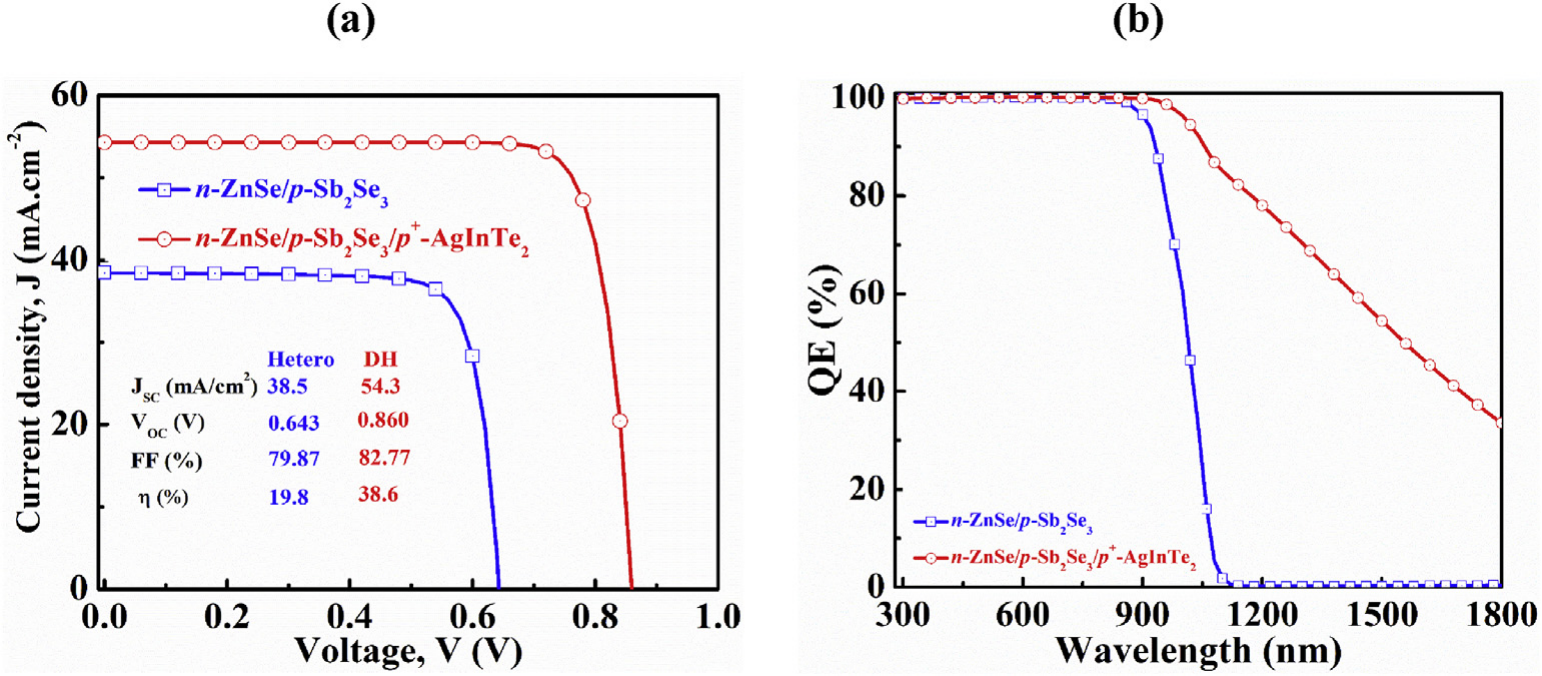
Simulated (a) light J-V and (b) QE curves of Sb_2_Se_3_-based single and dual-heterojunction solar cells.

The current-voltage curves under illumination and quantum efficiency for Sb_2_Se_3_-based single *n-p* heterojunction and double *n-p-p*^*+*^ heterojunction solar cells are displayed in Fig. 6a and b, respectively. It is evident from Figure 6a that the installation of a p + -AgInTe_2_ layer revamps the cell performance substantially. The reason behind such an overshoot can be demonstrated with the TSA photon upconversion process where sub-band gap photons are occupied by the Urbach states (Mostaque et al., 2022; Mondal et al., 2021; Kuddus et al., 2021a). These low energy sub-band gap photons take part in generating electron-hole pairs contributing to the photocurrent (Yablonovitch et al., 1982; Wu and Williams, 1983). The degree of current enhancement and hence QE depend not only on Urbach energy but also on the band gap of the material. The generation of high built-in potential at the Sb_2_Se_3_/AgInTe_2_ interface further enhances the value of *V*_*OC*_ (Hossain et al., 2021b). As a result, the efficiency has remarkably improved due to the addition of AgInTe_2_ as bottom layer. Figure 6b reveals that the QE in *n-p* heterojunction structure falls to 0% at the wavelength of 1100 nm whereas, it displays about 40% corresponding QE in the proposed *n-p-p*^*+*^ structure with the inclusion of AgInTe_2_ BSF layer. The QE seems to theoretically remain around 30% even for 1800 nm wavelength. This finding indicates that AgInTe_2_ could be a promising candidate for improving the PCE of Sb_2_Se_3_-based solar cells in near future.

## 4 Conclusion

The present work demonstrates the numerical simulation of Sb_2_Se_3_-based *n*-ZnSe/*p*-Sb_2_Se_3_/*p*^*+*^-AgInTe_2_ dual-heterojunction solar cell using SCAPS-1D software by incorporating interface defects and varying thickness, doping concentration and bulk defects in each layer. The simulation reveals that the incorporation of AgInTe_2_ which also serves as a bottom absorber layer can collect longer wavelength photons through TSA two-steps photon upconversion process, resulting a considerable enhancement in photocurrent. The optimized performance considering 0.1 μm thick *n*-ZnSe window and 1.0 μm *p*-Sb_2_Se_3_ absorber provides an efficiency of 19.8% that shoots up to 38.6% with *J*_*SC*_ of 54.3 mA/cm^2^, *V*_*OC*_ of 0.860 V and *FF* of 82.77%, respectively owing to the incorporation of only a 0.5 μm AgInTe_2_ BSF layer. Overall, the study suggests that AgInTe_2_ compound as a bottom layer displays strong potential for the enhancement of the efficiency of Sb_2_Se_3_-based solar cells in future.

## Declarations

### Author contribution statement

Bipanko Kumar Mondal: Conceived and designed the experiments; Performed the experiments; Analyzed and interpreted the data; Wrote the paper.

Shaikh Khaled Mostaque: Analyzed and interpreted the data; Wrote the paper.

Jaker Hossain: Conceived and designed the experiments; Contributed reagents, materials, analysis tools or data; Wrote the paper.

### Funding statement

This research did not receive any specific grant from funding agencies in the public, commercial, or not-for-profit sectors.

### Data availability statement

Data included in article/supplementary material/referenced in article.

### Declaration of interests statement

The authors declare no conflict of interest.

### Additional information

No additional information is available for this paper.
